# *Caenorhabditis briggsae* Recombinant Inbred Line Genotypes Reveal Inter-Strain Incompatibility and the Evolution of Recombination

**DOI:** 10.1371/journal.pgen.1002174

**Published:** 2011-07-14

**Authors:** Joseph A. Ross, Daniel C. Koboldt, Julia E. Staisch, Helen M. Chamberlin, Bhagwati P. Gupta, Raymond D. Miller, Scott E. Baird, Eric S. Haag

**Affiliations:** 1 Department of Biology, University of Maryland, College Park, Maryland, United States of America; 2 Department of Genetics, Washington University School of Medicine, St. Louis, Missouri, United States of America; 3 Department of Molecular Genetics, Ohio State University, Columbus, Ohio, United States of America; 4 Department of Biology, McMaster University, Hamilton, Canada; 5 Department of Biological Sciences, Wright State University, Dayton, Ohio, United States of America; Princeton University, United States of America

## Abstract

The nematode *Caenorhabditis briggsae* is an emerging model organism that allows evolutionary comparisons with *C. elegans* and exploration of its own unique biological attributes. To produce a high-resolution *C. briggsae* recombination map, recombinant inbred lines were generated from reciprocal crosses between two strains and genotyped at over 1,000 loci. A second set of recombinant inbred lines involving a third strain was also genotyped at lower resolution. The resulting recombination maps exhibit discrete domains of high and low recombination, as in *C. elegans*, indicating these are a general feature of Caenorhabditis species. The proportion of a chromosome's physical size occupied by the central, low-recombination domain is highly correlated between species. However, the *C. briggsae* intra-species comparison reveals striking variation in the distribution of recombination between domains. Hybrid lines made with the more divergent pair of strains also exhibit pervasive marker transmission ratio distortion, evidence of selection acting on hybrid genotypes. The strongest effect, on chromosome III, is explained by a developmental delay phenotype exhibited by some hybrid F2 animals. In addition, on chromosomes IV and V, cross direction-specific biases towards one parental genotype suggest the existence of cytonuclear epistatic interactions. These interactions are discussed in relation to surprising mitochondrial genome polymorphism in *C. briggsae*, evidence that the two strains diverged in allopatry, the potential for local adaptation, and the evolution of Dobzhansky-Muller incompatibilities. The genetic and genomic resources resulting from this work will support future efforts to understand inter-strain divergence as well as facilitate studies of gene function, natural variation, and the evolution of recombination in Caenorhabditis nematodes.

## Author Summary

The nematode *Caenorhabditis briggsae* is increasingly used for comparisons with its more famous relative, *C. elegans*. To improve genomic resources for *C. briggsae*, we created two sets of inbred lines derived from crosses between diverged *C. briggsae* strains. High-throughput genotyping of these has improved the resolution of the recombination map and genome assembly. It also allows detailed comparisons of recombination both within and between species. Unexpectedly, we found that alleles from one parental strain were much more likely to be fixed on three of the six chromosomes in one of the sets of lines. One of these biases is caused by a pronounced developmental delay in F2 progeny that is seen in both reciprocal crosses, whereas the other two manifest in only one of the two cross directions. This indicates that the parental strains have diverged in both nuclear and nuclear-cytoplasmic interactions, either because of local adaptation or restricted gene flow across much of the genome.

## Introduction

Caenorhabditis nematodes, first described over one hundred years ago [1], are easily cultured and have been employed since the 1960s as model organisms in a number of fields. *C. briggsae* exhibits many features desirable of a genetic model organism: a self-fertilizing hermaphrodite, presence of rare males for genetic crosses, and broods of hundreds that reach sexual maturity in a few days [2]. Sydney Brenner initially touted *C. briggsae* as the model system of choice for studying the genetic basis of cellular development, although he eventually championed the now-famous *C. elegans* [3], [4]. The many similarities between *C. briggsae* and *C. elegans* [5] led to confusion as to which strains belonged to which species until 1977 [6], and it seems *C. briggsae* could easily have been the more widely-studied species today.

More recent reports have revealed key ways in which *C. briggsae* differs from *C. elegans*. For example, genetic and phylogenetic studies have demonstrated that *C. elegans* and *C. briggsae* independently evolved self-fertile hermaphroditism by means of distinct genetic mechanisms [7]–[11]. Surprising differences also exist in their early embryonic patterning [12] and anatomy of the excretory system [13], [14].

*C. elegans* and *C. briggsae* also differ in their phylogeography. Global sampling of natural isolates suggests near-panmixia among *C. elegans* populations [15]–[24], while strong latitudinal population structure exists in *C. briggsae* [17], [25]–[28]. Thus, while sharing reproductive mode and cosmopolitan distribution, *C. elegans* and *C. briggsae* appear to migrate and interbreed at different rates, and as a result have differing levels of species-wide genetic variation [18], [26]. Despite its minimal population structure, however, *C. elegans* harbors a polymorphic (and potentially selfish) incompatibility locus that causes hybrid lethality [29]. Evidence of outbreeding depression in *C. briggsae* has also been noted [17], though its genetic structure is unknown.

The greater genetic and phenotypic variation in *C. briggsae* makes it useful for mapping loci affecting various traits, such as male tail development, vulva cell fate, and fecundity [17], [27], [30]–[33], and refutes an early criticism of Caenorhabditis “that the animal has few morphological and behavioral traits” [4]. Some of these studies sought to identify ecological correlates of phylogeography, such as temperature, that might explain the diversity exhibited among *C. briggsae* strains. However, no such correlations between geography, genotype, and phenotype have been made for *C. elegans*, and they might not exist [34], [35]. Thus, *C. briggsae* can be both a critical companion species for comparative analysis with *C. elegans* and also a potentially better choice for studies investigating the genetic architecture of ecological adaptation in nature. Both of these roles demonstrate the value of continued development of *C. briggsae* as a model system.

Research on *C. briggsae* has enjoyed a recent surge in popularity [e.g. 8], [17], [26], [31], [36] since its genome was sequenced [37]. The last decade has seen improvement of the genetic and genomic research tools available [37]–[40], but they still lag behind those for *C. elegans*. Initially motivated by a desire to improve *C. briggsae* as a genetic system, we produced and genotyped advanced-intercross recombinant inbred lines. Such cross designs have been employed in other species [15], [41]–[43] and are particularly useful for expanding genetic maps [44]. Such an improved map allows precise comparisons of recombination landscapes for homologous chromosomes. *C. briggsae* is similar to *C. elegans* in a number of genetic and population genetic characteristics (*e.g.* low effective population size [24], [28], frequent self-fertilization, equivalent genome size [37], and strong crossover interference [38]). This raises the possibility that variation in recombination rate might contribute to their different levels of DNA polymorphism [18], [26]. Previous studies suggest that a general chromosome-wide pattern of recombination rate domains is conserved between the two species [15], [38]. However, the low resolution and sparse density of genetic markers in the previous *C. briggsae* genetic map diminish the accuracy of such a comparison. Intra-species variation of recombination rates among wild-type strains has been examined in *C. elegans* [15], [45]; a comparison of intra-species (*C. briggsae*) and inter-species (*C. elegans* – *C. briggsae*) recombination profiles might reveal how recombination rates evolve over timescales as small as hundreds of thousands of years.

The stereotyped and discrete domains of recombination common to Caenorhabditis [15], [38] also aid identification of correlates of change in recombination rate. For example, inversions alter recombination when heterozygous, often suppressing (but not always absolutely) recombination within them [46]–[50] and increasing it around them [51]. Such rearrangements are also thought to contribute to adaptation and speciation [52]–[56]. A comparison of intraspecific genetic maps could clarify the relationship between inversions, adaptation and speciation in different populations.

**Figure 1:**
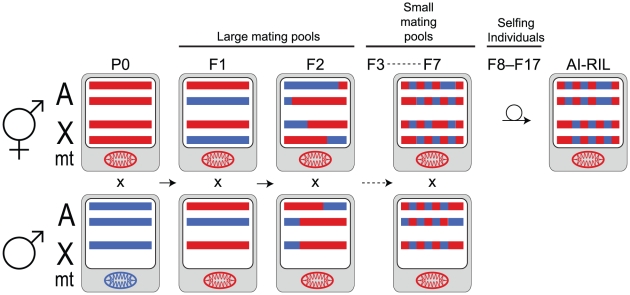
AI-RIL cross scheme. An autosome pair (A) and the X chromosome pair (X) are depicted as horizontal bars for each parent in selected generations. Males have a single maternally-inherited X chromosome. Chromosomes are shown on a white background, representing the nucleus. The oval mitochondrial genome (mt) is shown on a gray background, representing the cytoplasm. P0 strains AF16 (red) and HK104 (blue) were crossed in both directions (AF16 male×HK104 hermaphrodite shown here). The F1 hybrids are mated with siblings; sib-mating continues through the F7. F8 hermaphrodites were selfed to produce an F9 generation; continual selfing through F17 was employed to inbreed the lines. Color-coded blocks depict the increase of haplotype breakpoints and homozygosity with generation. Under neutral expectations, two thirds of the X chromosomes in the lines will be contributed by the hermaphrodite. The hermaphrodite (i.e. oocyte) parent also contributes its mitochondria to the AI-RIL, although heteroplasmy has been observed in *C. briggsae* [27], [112].

In this study, we produced and genotyped two sets of *C. briggsae* recombinant inbred lines (RIL). One set was generated from the strains AF16 and HK104 using an advanced-intercross design (AI-RIL; Figure 1). Roughly half of these AI-RIL were established in one cross direction (AF16×HK104, where the first strain listed provides the male, by convention) and half in the other (HK104×AF16). [Note: when discussing both subsets of AI-RIL without respect to cross polarity, the notation “AF16/HK104” will be used]. The second set of RIL was generated from the strains AF16 and VT847 using an F2 cross scheme. The linkage maps derived from these two sets of RIL are suited for revealing differences in relative recombination rates. We also used the sets of RIL to detect selection occurring on hybrid genotypes and to identify inter-strain genetic incompatibilities, revealing the potential utility of *C. briggsae* for studying the process of incipient speciation in a highly selfing species.

## Results

### SNP Genotype Data Set

The first-generation *C. briggsae* genetic map was produced by RIL generated by the selfing of F2 founders [38]. *C. elegans* chromosomes generally experience one recombination event per meiosis [57]. Assuming that *C. briggsae* is similar, F2 RIL contain few recombination breakpoints per chromosome, limiting their utility for making genetic maps [38]. We therefore created a set of advanced-intercross recombinant inbred lines (AI-RIL) for *C. briggsae* in order to improve the genetic map. We used six generations of mating prior to ten generations of selfing to decrease the size of haplotype blocks in the AI-RIL (Figure 1). The parental strains were *C. briggsae* AF16, the standard laboratory strain from India whose genome has been sequenced [37], and HK104, a divergent Japanese strain already used for SNP discovery and mapping [7], [16], [39], [58]. AF16 and HK104 are members of distinct tropical and temperate clades of *C. briggsae* [28], respectively, that diverged roughly 90,000 years ago [26].

180 AI-RIL and the parental strains were genotyped at 1,536 single nucleotide polymorphism (SNP) markers. 167 AI-RIL and 1,032 SNP markers passed quality control thresholds and inspections (Materials and Methods), resulting in 172,344 genotype calls for the AI-RIL (Table S1). After exclusion of lines apparently heterozygous at many markers (Materials and Methods), only three heterozygous genotype calls remain in the final genotype data set. The remaining genotypes were homozygous for one of the parental strains (67,286 AF16/AF16; 105,055 HK104/HK104). Homozygosity of the parental strains at each marker was confirmed directly (Table S1).

89 F2 RIL were produced by repeatedly selfing the offspring of VT847×AF16 F1 hybrids. VT847 is a *C. briggsae* isolate from Hawaii [30], part of the same clade of tropical isolates as AF16 [17]. These RIL were genotyped at the same 1,536 SNPs. Mostly because many of these SNPs are monomorphic between the parental strains, only 209 markers passed quality control. Again, the vast majority of genotype calls were homozygous for one of the parental strains (9,344 AF16/AF16; 9,184 VT847/VT847); 50 calls were heterozygous (Table S1, but see Materials and Methods). 132 markers were successfully genotyped in both sets of RIL.

### Construction of Genetic Maps

**Figure 2:**
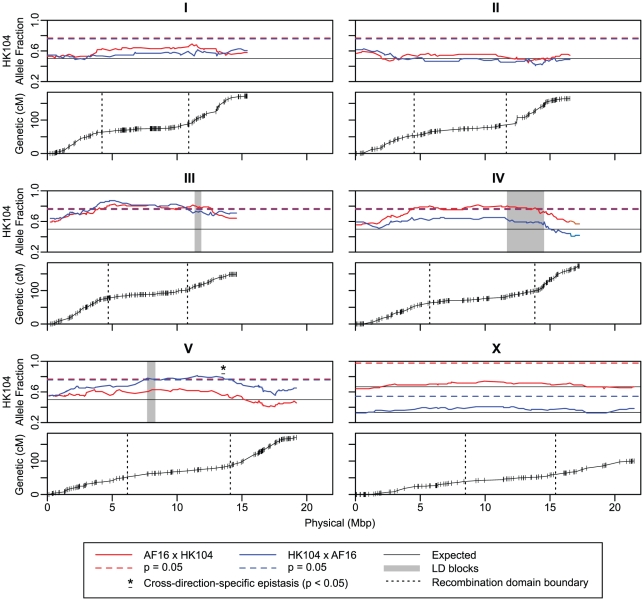
AI-RIL allele fraction and Marey plots. The upper panel for each of the six nuclear chromosomes depicts the fraction of AI-RIL fixed for the HK104 allele (y axis) at each genetic marker. The markers are ordered by physical position in the chromosome assembly (Mbp) on the x axis. For all chromosomes, the blue and red solid lines indicate the allele fraction at each marker for the HK104×AF16 and the AF16×HK104 cross directions, respectively. For autosomes, a single solid black horizontal line indicates the neutral-expected allele fraction of 0.5, and the dashed red and blue lines indicate the allele fraction at which deviation from the expected value becomes significant for each cross direction at p = 0.05 (chi-square, Bonferroni-corrected by genome-wide effective number of tests). For ChrX, the distinct cross-specific expectations (solid black lines) and significance thresholds (dashed red and blue lines) are both given. Only HK104-biased thresholds are depicted, as the allele fraction values are never significantly AF16-biased. Three shaded areas show markers comprising interchromosomal LD blocks of high D′ value (Figure 6). The asterisk and horizontal bar indicate the positions of three markers on ChrV whose allele fractions differ significantly from each other by cross direction. The lower panel for each chromosome depicts the relationship between physical position and genetic position of each marker; the y axis is genetic position in the linkage group in centimorgans (cM). Positions of genotyped SNP markers are shown as vertical lines. Vertical dotted lines depict the positions of the arm-center recombination rate domain boundaries estimated by linear regression.

Genetic maps of the five autosomes and X chromosome comprising the nuclear genome were estimated *de novo* from the final AF16/HK104 AI-RIL SNP genotype data set. Marker compositions and lengths of the maps are given in Table 1. The expanded AI-RIL genetic maps for autosomes range from 148.6 to 173.2 centimorgans (cM) in cumulative length; the X chromosome map length is 100.0 cM. The new *C. briggsae* genome assembly (see below) inferred from the genetic map allowed us to plot the recombination rate as a function of physical position (Marey maps; [59], Figure 2). This reveals the presence on each chromosome of small tip domains and larger central domains that host less recombination compared to the chromosome “arm” domains (Caenorhabditis chromosomes are holocentric [60]). As previously found in *C. elegans* and *C. briggsae*, the X chromosome domain boundaries are qualitatively less evident than those of the autosomes [15], [38].

**Table 1:** Linkage map and genome assembly statistics.

	Cb4 (this study)				Cb3 ([38])			
Chr	Map Length^a^(cM)	Sequence(bp)	sctgs	SNPs	Map Length^a^^,^ b(cM)	Sequence(bp)	sctgs	SNPs
I	171.7	15451279	48	180	88.9	11272543	24	40
II	164.5	16622654	46	153	106.2	14511075	20	45
III	148.6	14574751	42	177	94.3	13541962	27	47
IV	173.2	17479539	60	213	99.2	15287474	29	56
V	170.6	19490057	52	177	111.2	16001401	28	49
X	100.0	21537770	29	131	88.3	20606332	18	53
Chr*_random	-	287801	8	1	-	9929549	30	-
Unassembled	-	2913214	353	-	-	7268690	431	-
Total	928.6	108357065	638	1032	588.1	108419026	607	290

For each linkage group (chromosome), the map length (in centimorgans, cM), genome sequence contained in the chromosome assembly (not including gaps), number of supercontigs (sctgs) in the chromosome assembly, and number of SNP markers genotyped are given. Values for the current (cb4) and previous (cb3) assemblies are provided for comparison. The Chr*_random assemblies contain any supercontigs that are mapped to chromosomes but cannot be ordered relative to other supercontigs in the chromosome assemblies. The unassembled chromosome contains all remaining supercontigs, which did not contain genotyped markers and so were not mapped to linkage groups.

a Cumulative genetic length, such that all recombination events evident on all chromosomes in a RIL set are considered to have occurred in a single meiosis.

b We re-estimated the cb3 genetic maps using the data from [38] in order to convert the map lengths, which had been reported as per-meiosis, to the cumulative lengths shown.

Of the 1,031 *C. briggsae* SNPs used to produce chromosome assemblies (one marker was genetically mapped but not used in the chromosome assemblies), only 443 genetic intervals are defined, owing to the complete linkage of a number of SNPs. The average size of an interval is 101.3 kbp, with median size 43.8 kbp and maximum of 1.45 Mbp. The average marker spacing is 2.1 cM, with median spacing 1.2 cM and a maximum of 18.7 cM. We note that these values represent cumulative genetic distance defined for the AI-RIL, not per-meiosis distances. Normalizing each linkage group to the expected per-meiosis map length of 50 cM, the average marker spacing becomes 0.6 cM. The genotypes of the VT847×AF16 RIL were also used to estimate *de novo* genetic maps; the genetic positions of markers and the genotypes of the RIL are given in Table S1. The estimated genetic maps for autosomes range in length from 82.1–110.6 cM; the X map is 43.0 cM.

The number of autosomal recombination breakpoints captured by the *C. briggsae* AF16/HK104 AI-RIL constructed for this study ranged from zero to six with an average of 1.59 (Table 2), less than might be expected given the cross design. Nevertheless, in the AI-RIL, autosomes exhibit almost twice as many evident recombination events compared to our F2 RIL and to the F2 RIL used to create the previous *C. briggsae* genetic map version [38] (Table 2). The AI-RIL and F2 RIL reported here also almost double the observed number of recombination events on the X chromosome.

**Table 2:** Recombination breakpoints in RIL.

Chr	AF16/HK104 AI-RIL	VT847×AF16 F2 RIL	HK104×AF16 F2 RIL^a^
I	1.63	0.88	0.69
II	1.57	0.74	0.85
III	1.44	0.83	0.70
IV	1.66	0.89	0.76
V	1.63	0.93	0.78
X	0.96	0.42	0.61

For our AI-RIL, our F2 RIL, and the F2 RIL used to produce the previous *C. briggsae* genetic map cb3, the number of observed recombination breakpoints, averaged across the number of RIL, is given for each chromosome. The advanced-intercross design leads to greater breakpoint capture than in F2 RIL.

a Breakpoint counts for the F2 RIL from [38] were obtained by re-estimating the cb3 genetic maps using their genotype data with Map Manager QTXb20 and averaging across the 93 lines they genotyped (Materials and Methods).

### *Caenorhabditis briggsae* Genome Assembly cb4

The 1,032 genetically mapped markers represent a four-fold increase in the number of markers used to produce *C. briggsae* genome assembly version cb3 [38] (Table 1). Combined with the increased number of recombination breakpoints afforded by the AI-RIL, the new genetic map facilitated the incorporation of unplaced sequence supercontigs, orientation of previously unoriented supercontigs, and identification and resolution of some existing assembly errors. Table 1 provides statistics on the new assembly, version cb4. Most notably, we have confidently ordered an additional 14 Mbp of sequence (13% of the genome), representing a 2.5-fold reduction in the amount of sequence unassigned to chromosomes and a 34-fold reduction in the amount of sequence unable to be ordered within chromosome assemblies. Importantly, 1.8 Mbp of sequence contained on 15 supercontigs has changed chromosomal assignment from cb3 to cb4. We also orient sequence contigs comprising 21 Mbp (20% of the genome). Additional details of the assembly are available in Text S1.

### Evidence for Inter- and Intra-Species Chromosomal Rearrangements

**Figure 3:**
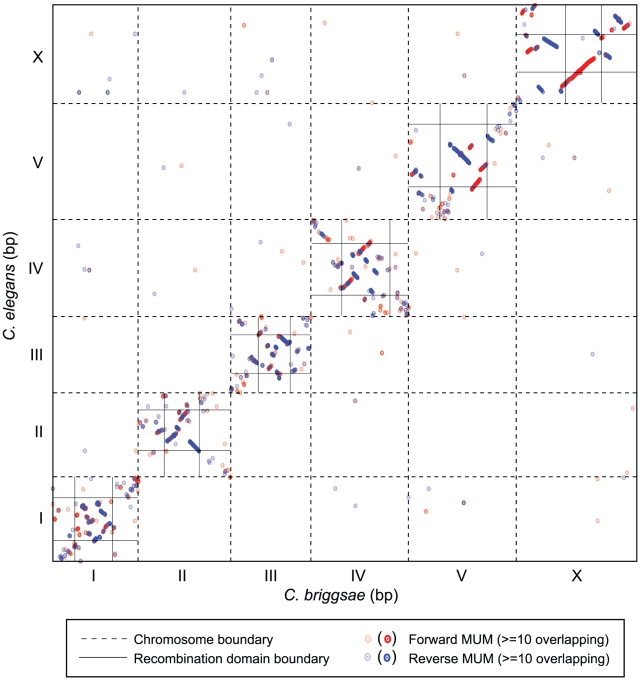
Chromosome synteny between *C. elegans* and *C. briggsae*. The concatenated nucleotide sequences of the *C. elegans* chromosomes were aligned to the concatenated *C. briggsae* genome. Dashed lines indicate the breakpoints between individual chromosome sequences. Maximal unique matches (MUMs) are depicted as dots: red indicates forward matches; blue indicates reverse matches. The lightest colors indicate a single MUM; the darkest colors indicate > = 10 adjacent MUMs that are not spatially resolved here. Vertical and horizontal black lines in the self diagonal indicate the positions of the recombination domain boundaries for *C. elegans* [15] and for *C. briggsae*. Extensive synteny is evident in the low-recombining central domains of all chromosomes; synteny is not apparent when comparing the chromosome arms between species.

With an improved genome assembly, we re-evaluated the extent of chromosomal synteny between *C. elegans* and *C. briggsae* using a genome-wide plot of nucleotide conservation. By identifying only maximal unique matches (MUMs) in each comparison sequence, orthologous coding regions are predominantly identified (Figure 3, by comparison with plots of MUMs using translated nucleotide sequence, not shown). Extensive matches exist in the self-diagonal (comparisons between homologous chromosomes of *C. elegans* and *C. briggsae*), but relatively few off-diagonal (interchromosomal) MUMs are apparent. The center domains of the autosomes have extensive colinearity in MUMs, while synteny in the arms is much less apparent. Although syntenic blocks on the X are larger and comprise a larger proportion of the chromosome than on autosomes, the order of blocks on the X nevertheless differs between the species.

While interspecies inversions and translocations are evident in these chromosomal plots, the presence and extent of polymorphic inversions among *C. briggsae* strains is unknown. By comparing our AF16/HK104 AI-RIL linkage maps with the VT847×AF16 F2 RIL linkage maps, we sought evidence for such inversions. Because heterozygous inversions present in hybrids should suppress recombination [46], inversions are expected to manifest genetically as blocks of markers that are recombinant with each other in one linkage map and nonrecombinant in the other. For all 132 SNPs common to both genetic maps, we ordered the SNPs based on physical assembly position and then identified blocks of markers that exhibit this genetic signature of inversion (Table S2).

Twenty-one blocks of markers are nonrecombinant in the F2 RIL but resolved in the AI-RIL; in the AI-RIL, four nonrecombinant blocks are resolved in the F2 RIL. Most of the former are expected due to the overall shorter F2 RIL map, whereas the latter might be enriched for true recombination suppressors. For example, ChrIV markers cbv19538 and cb58228 acted as a point in the AI-RIL genetic map, but were 1 cM apart in the F2 RIL map of ChrIV normalized to 50 cM. These markers reside in high recombination arm B of ChrIV, where the normalized breakpoint density in the AI-RIL map is 5.66 breakpoints/cM. We thus expect to see 5.03 breakpoints between them, averaged over the 89 F2 RIL. Assuming that the breakpoints are Poisson-distributed with an expected value of 5.03, the observed value of zero is significantly different (p = 0.006). When Bonferroni-corrected for multiple tests, the genetic distance between these markers in the F2 RIL remains significant (p<0.05).

### Inter-Species Variation in Recombination

We estimated the physical and genetic size and recombination rate of each domain (Table 3). To allow comparisons between maps of different overall lengths, the recombination rates in the AI-RIL were normalized by adjusting the map length of each chromosome to the expected per-meiosis length of 50 cM (see Materials and Methods). Low synteny in the chromosome arms (Figure 3) precludes meaningful direct comparisons of arms between species. We therefore refer to the arms of *C. briggsae* chromosomes as “A” and “B” rather than “L” (left) and “R” (right) to prevent inappropriate inference of homology, and we compare arm attributes between *C. briggsae* and *C. elegans* using ratios of lengths and rates from one arm to the other. The homology of center domains is not ambiguous, so their values can be compared directly.

**Table 3:** Recombination domain comparison between *C. briggsae* and *C. elegans*.

		*C. briggsae* AI-RIL Recombination Rate Domains											
Chr		Tip		A		Center		B		Tip		Total	A-BRatio
I	Mbp	0.37	2.43%	3.84	24.83%	6.68	43.26%	4.31	27.91%	0.24	1.58%	15.45	1.12
	cM^a^	0	0	18.63	37.26%	7.06	14.11%	24.31	48.63%	0	0	50	1.30
	Rate(cM/Mbp)	0	-	4.86	-	1.06	-	5.64	-	0	-	-	1.16
II	Mbp	0.09	0.56%	4.44	26.68%	7.11	42.74%	4.90	29.50%	0.09	0.52%	16.62	1.11
	cM^a^	0	0	16.48	32.96%	9.61	19.23%	23.91	47.81%	0	0	50	1.45
	Rate(cM/Mbp)	0	-	3.72	-	1.35	-	4.87	-	0	-	-	1.31
III	Mbp	0.36	2.47%	4.34	29.74%	6.11	41.89%	3.29	22.56%	0.49	3.35%	14.57	1.32
	cM^a^	0	0	25.98	51.95%	8.89	17.78%	15.13	30.27%	0	0	50	1.72
	Rate(cM/Mbp)	0	-	5.99	-	1.46	-	4.60	-	0	-	-	1.30
IV	Mbp	0.46	2.64%	5.27	30.12%	8.11	46.37%	3.31	18.92%	0.34	1.95%	17.48	1.59
	cM^a^	0	0	18.10	36.20%	10.78	21.56%	21.12	42.24%	0	0	50	1.17
	Rate(cM/Mbp)	0	-	3.44	-	1.33	-	6.38	-	0	-	-	1.86
V	Mbp	0.12	0.64%	6.05	31.05%	7.93	40.68%	5.11	26.20%	0.28	1.44%	19.49	1.19
	cM^a^	0	0	15.38	30.76%	10.13	20.27%	24.49	48.98%	0	0	50	1.59
	Rate(cM/Mbp)	0	-	2.54	-	1.28	-	4.80	-	0	-	-	1.89
X	Mbp	0.79	3.66%	7.70	35.73%	6.95	32.25%	5.93	27.54%	0.18	0.82%	21.54	1.30
	cM^a^	0	0	19.57	39.15%	10.40	20.80%	20.03	40.06%	0	0	50	1.02
	Rate(cM/Mbp)	0	-	2.54	-	1.50	-	3.38	-	0	-	-	1.33

		*C. elegans* AI-RIL Recombination Rate Domains^b^											
Chr		Tip		L		Center		R		Tip		Total	L-RRatio
I	Mbp	0.53	3.50%	3.33	22.10%	7.18	47.65%	3.84	25.44%	0.20	1.31%	15.07	1.15
	cM^a^	0	0	12.06	24.11%	11.09	22.18%	26.86	53.71%	0	0	50	2.23
	Rate(cM/Mbp)	0	-	3.62	-	1.54	-	7.00	-	0	-	-	1.93
II	Mbp	0.31	2.00%	4.57	29.93%	7.14	46.74%	2.59	16.94%	0.67	4.39%	15.28	1.77
	cM^a^	0	0	19.28	38.55%	10.38	20.75%	20.35	40.70%	0	0	50	1.06
	Rate(cM/Mbp)	0	-	4.22	-	1.45	-	7.86	-	0	-	-	1.86
III	Mbp	0.49	3.58%	3.23	23.42%	6.62	48.01%	2.88	20.87%	0.57	4.11%	13.78	1.12
	cM^a^	0	0	22.45	44.90%	8.98	17.96%	18.57	37.14%	0	0	50	1.21
	Rate(cM/Mbp)	0	-	6.95	-	1.36	-	6.45	-	0	-	-	1.08
IV	Mbp	0.72	4.12%	3.18	18.15%	9.07	51.87%	3.74	21.39%	0.78	4.47%	17.49	1.18
	cM^a^	0	0	24.58	49.17%	11.89	23.77%	13.53	27.06%	0	0	50	1.82
	Rate(cM/Mbp)	0	-	7.74	-	1.31	-	3.62	-	0	-	-	2.14
V	Mbp	0.64	3.07%	5.25	25.11%	10.65	50.92%	3.79	18.10%	0.58	2.79%	20.92	1.39
	cM^a^	0	0	16.02	32.05%	14.48	28.97%	19.49	38.99%	0	0	50	1.22
	Rate(cM/Mbp)	0	-	3.05	-	1.36	-	5.15	-	0	-	-	1.69
X	Mbp	0.57	3.23%	5.57	31.41%	6.34	35.80%	3.94	22.22%	1.30	7.35%	17.72	1.41
	cM^a^	0	0	19.73	39.45%	11.30	22.60%	18.97	37.95%	0	0	50	1.04
	Rate(cM/Mbp)	0	-	3.54	-	1.78	-	4.82	-	0	-	-	1.36

For each of the five domains per chromosome, values of physical (Mbp) and genetic (cM) length and the recombination rate (cM/Mbp) are given (left column) as well as the percent of the total chromosome physical and genetic length occupied by each domain (right column). The genetic lengths of the three major domains (L, Center, R for *C. elegans*; A, Center, B for *C. briggsae*) were normalized using a single factor per chromosome so that the map lengths of the three domains sum to 50 cM. The second column from the right contains totals of the physical and normalized genetic map lengths. The column at far right contains the fold-change between the sizes and rates of the two chromosome arms.

a Normalized to yield linkage groups of 50 cM total genetic length.

b Data from [15], except genetic lengths estimated and normalized as for *C. briggsae* based on recombination domain genetic lengths kindly provided by M. Rockman (unpublished data).

-: Not Applicable.

The center domains occupy more than a third of the physical length of each autosome (Table 3). However, they are relatively smaller in *C. briggsae* (comprising 40–46% of the total chromosome length in *C. briggsae* vs. 47–52% in *C. elegans* [15]). On the X chromosome in both species, the center domain occupies closer to a third of the chromosome length. Compared to their physical lengths, the genetic lengths of central domains are short compared to the arms in both species (but they still exhibit variation, *e.g.* ChrI, ChrV). Tip domains tend to occupy larger proportions of the chromosome length in *C. elegans* than *C. briggsae*. The absence of tip domains on the B arms of *C. briggsae* ChrII and ChrV could represent real diversity or be due to poor marker coverage in those regions.

The ratios of arm physical sizes are similar, ranging from 1.11–1.59 in *C. briggsae* and 1.12–1.77 in *C. elegans* (Table 3). However, arm genetic lengths vary more between species. For example, the ratio of genetic lengths of the two ChrII arms is 1.45 in *C. briggsae*, but 1.06 in *C. elegans*. Strikingly, genetic and physical length ratios do not always correlate. *C. briggsae* ChrIV arms have the largest asymmetry in physical length (1.59-fold) but the smallest in genetic length (1.17-fold). The opposite pattern is seen in *C. elegans*, whose ChrIV arms have a physical length ratio of 1.18 but a genetic length ratio of 1.82. Arm ratios for the X are similar between the two species.

Chromosomal attributes that dictate the sizes or boundaries of recombination domains are expected to co-vary in the two species. To identify candidate attributes, we compared three characteristics of homologous *C. elegans* and *C. briggsae* center domains: their genetic length, physical length and proportion of the chromosome physical length. We also examined the degree of asymmetry in arm pairs as measured by the ratios of their genetic and physical lengths. Of these, the fraction of the total physical chromosome length occupied by a given central domain in one species was the most predictive of the state for the homolog in the other (R^2^ = 0.8253).

### Intra-Species Chromosomal Recombination Domain Comparisons

**Figure 4:**
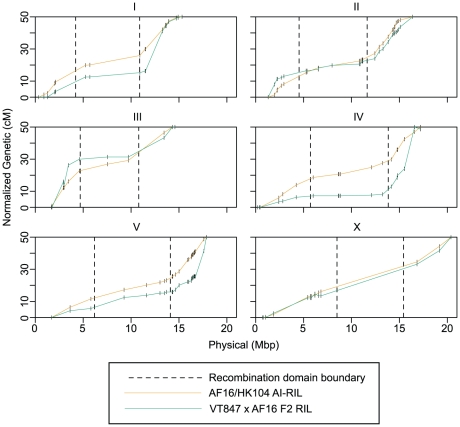
Intra-species recombination rate comparison. The Marey maps for the AF16/HK104 AI-RIL and the VT847×AF16 F2 RIL are provided for one chromosome per panel. Only markers successfully typed in both RIL sets are plotted, shown as short vertical lines. The map positions of these markers were normalized to produce 50 cM maps. Vertical dashed lines indicate the positions of recombination domain boundaries defined by the AI-RIL genetic map. Line segments with negative slopes (A arm domains of ChrIII and ChrX and B arm terminus of ChrIV) are blocks of markers with inverted genetic order in the AI-RIL and F2 RIL (Table S2).

To identify variation in the recombination domains on a shorter time scale, we compared their characteristics in the AF16/HK104 AI-RIL and VT847×AF16 F2 recombination maps (Figure 4). As the low marker density of the F2 VT847-based map precludes precise *de novo* determination of recombination domain boundaries, we used the boundaries determined for the AI-RIL for both maps (visual inspection of the F2 RIL Marey maps, Figure 4, indicates this is reasonable). The comparison reveals two ways in which apparent recombination rates vary across a given chromosome (Figure 4). First, while the genetic lengths of the two arm domains of a given autosome are generally symmetrical in the AF16/HK104 map (fold-change range 1.17–1.72), observed recombination is often heavily biased to one arm in the VT847×AF16 maps (fold-change range 1.41–7.09). Second, the genetic lengths of the center domains can differ between AF16/HK104 and VT847×AF16 (for ChrIII and ChrIV, over two-fold). Thus, the Marey map curves visibly differ in the two maps for ChrI, ChrIII, ChrIV, and ChrV.

### Marker Transmission Ratio Distortion in Hybrid Lines

In the crossing scheme used to produce the AI-RIL, each parental strain is expected to contribute half of the alleles at any autosomal locus; for ChrX, two-thirds of lines are expected to fix the allele of the hermaphrodite parent in the original cross (Figure 1). Deviation from the neutral-expected allele fraction value is called marker transmission ratio distortion (MTRD) and can indicate the action of selection on specific hybrid genotypes. We plotted the relationship between the proportion of lines fixed for the HK104 allele and the physical position of each marker in order to identify departures from the neutral expectations (Figure 2). For ChrI, ChrII and ChrX, in neither cross direction does allele fraction significantly deviate from expected. However, for markers on the remaining autosomes, significant MTRD towards the HK104 parental allele was common. On ChrIV and ChrV, significant departure from the expected allele fraction value occurred only in one cross direction. On ChrIV, the AF16×HK104 AI-RIL were biased (maximum allele fraction = 0.81; 7.3 Mbp significantly biased); on ChrV, the HK104×AF16 AI-RIL were biased (maximum allele fraction = 0.81; 7.4 Mbp significantly biased). We hypothesized that epistatic genetic interactions between one or more loci in the central recombination domains of ChrIV or ChrV and a factor dictated by cross direction produces the observed MTRD. To directly test for cross direction effects, we compared allele fractions between the crosses in these regions. For ChrV, the allele fraction values of three adjacent markers (Figure 2, asterisk) were significantly different (p<0.05 after Bonferroni correction), while no ChrIV markers met this standard.

The most extreme MTRD was on ChrIII. The majority of ChrIII markers were biased toward the HK104 allele in both cross directions (maximum allele fraction = 0.87; AF16×HK104: 8.2 Mbp and HK104×AF16: 7.6 Mbp significantly biased). Despite the MTRD, at no marker was the AF16 allele completely absent from the AI-RIL set. Line PB1149, which had the fewest number of AF16/AF16 calls (137 of 1,032), exhibits only six recombination breakpoints and is fixed for HK104 across all of ChrI, ChrII and ChrIII.

### ChrIII MTRD Is Associated with a Developmental Delay Phenotype

**Figure 5:**
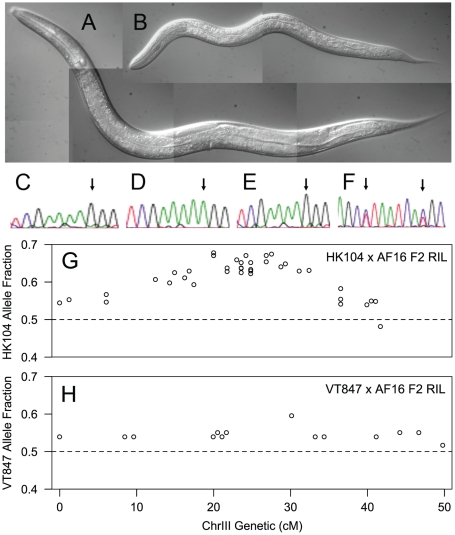
F2 slow-growth phenotype is linked to ChrIII. A,B) DIC micrographs, taken at the same magnification 48 hours after egg laying by an AF16/HK104 hybrid F1 hermaphrodite, of A) an adult non-delayed F2 and B) an L2 delayed F2 sibling. C–F) Sequence traces from *Cbr-egl-5* (CBG0023, ChrIII:12.2 Mbp) amplification products derived from pools of C) 50 AF16 individuals, D) 50 HK104 individuals and E) 50 delayed F2 hybrids show the biased segregation of AF16 alleles on ChrIII with the F2 delay phenotype. Arrows indicate the position of the polymorphic nucleotide in the TCGAAA[G/A]GG sequence. Similar results were obtained for *Cbr-glp-1* (CBG06809, ChrIII: 10.1 Mbp) (data not shown). F) A sequence trace shows two linked control SNPs from *Cbr-mab-20* (CBG22137, ChrI: 2.5 Mbp) amplification products derived from pools of 50 delayed F2 hybrids and demonstrates the unbiased segregation in delayed F2s of both AF16 and HK104 alleles on a different autosome. The arrows indicate the positions of the polymorphic nucleotides in the AGC[C/T]TAATCA[C/T]GC sequence. G,H) Comparison of ChrIII marker transmission ratios for two F2 RILs: G) HK104 allele fraction for all ChrIII markers mapped in the HK104×AF16 F2 RIL in [38] shows greater deviation from the expected value of 0.5 than seen in H), the VT847 allele fraction on ChrIII in the VT847×AF16 F2 RIL.

During production of the AI-RIL, we noticed that approximately 20% of F2 hybrids from crosses between AF16 and HK104 exhibit a pronounced developmental delay (Figure 5A and 5B; [17]). These delayed F2 take approximately four days to reach sexual maturity at 20°C, whereas P0s, F1s and most F2s reach sexual maturity in approximately three days. The delayed development of these F2s was associated with homozygosity for AF16 alleles in the central domain of ChrIII (Figure 5C–5F), consistent with the under-representation of AF16 alleles on ChrIII in the AI-RIL. The delay phenotype is reproducible in crosses between AF16 and HK104, but was not observed in VT847×AF16 F2 individuals during production of the F2 RIL (not shown). Furthermore, while a bias against AF16 alleles can be seen in the ChrIII genotypes of AF16×HK104 F2 RIL [38] (Figure 5G), no such bias is evident in the VT847×AF16 F2 RIL (Figure 5H).

### Genome-Wide Linkage Disequilibrium

**Figure 6:**
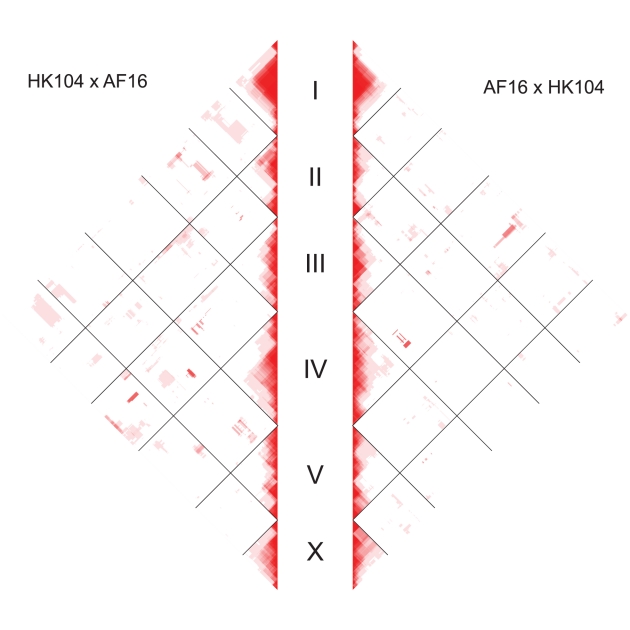
Genome-wide linkage disequilibrium by cross direction. The left panel shows LD values for the HK104×AF16 AI-RIL; the right panel shows LD for the AF16×HK104 AI-RIL. Linkage disequilibrium (D′) values for each pairwise comparison of markers were calculated and plotted. Marker spacing on the y axis is not scaled by physical or genetic distance; each marker occupies one unit space. Red indicates D′ = 1; shades of red indicate decreasing D′ values approaching 0 (white).

Characterization of interchromosomal linkage disequilibrium (LD) in the lines could identify co-adapted loci that might affect hybrid fitness, enhance the utility of the AI-RIL, and determine whether X-autosome epistatic interactions explain the cross direction-specific MTRD for ChrIV and ChrV described above. D′, a measure of LD that ranges from zero to one and normalizes D for overall allele frequencies [61], was employed as the metric here (Figure 6). Very few regions of high interchromosomal D′ values common to both cross directions were observed in this analysis. However, discrete blocks of high D′ present only in one cross direction are seen, including a block containing markers with interchromosomal D′ = 1. In this case, in the AF16×HK104 cross, AI-RIL whose genotypes are AF16/AF16 at cb22151 (ChrIII) are never also AF16/AF16 at cb4013 (ChrIV). However, D′ is calculated under the assumption of Hardy-Weinberg equilibrium, which might not be appropriate for inbred lines. Indeed, this correlation is not significant (chi-square, p = 0.058), most likely due to the strong HK104-biased allele frequencies of the AI-RIL set. Similarly, in the opposite cross direction, no gametic class frequencies are significantly different from expected based on the allele frequencies at these markers (chi-square, p = 0.773). It is nevertheless interesting to note that the same block of ChrIII markers interacts with a small region of ChrV in one cross direction and with ChrIV in the other (Figure 6). These three blocks on ChrIII, ChrIV and ChrV overlap with (but are much smaller than) regions of significant MTRD (the blocks are identified by shading in Figure 2).

## Discussion

### An Improved Genetic Map for *C. briggsae*

The previous *C. briggsae* genetic map was based on SNP genotyping of F2 RIL [38]. Because Caenorhabditis chromosomes generally experience one crossover event per meiosis [57], these RIL have very large haplotype blocks. While this did not hinder assignment of sequence supercontigs to linkage groups, it often prevented the supercontigs from being ordered and oriented within a chromosome [38]. The five additional generations of mating beyond F2 used to produce the AI-RIL (Figure 1) expanded the genetic map to 928.6 centimorgans total length, a 1.57-fold increase compared to the cb3 genetic map. In addition, we were able to substantially increase the map's resolution by more than tripling the number of scored SNPs (1,032) in almost twice (167) the number of inbred lines (Table 1). Our AI-RIL genetic map compares favorably with other contemporary maps in marker number (1,032) and density (0.6 cM average spacing when normalized to a 50 cM map length). Those recently estimated in the genera Bombyx, Apis, Nasonia, and Brassica contain between 1,000 and 2,000 markers, producing 0.3–2.05 cM average marker spacing [62]–[65].

Our map did not match the quality of the *C. elegans* AI-RIL-based genetic map [15], however. This map captured 3,629 recombination breakpoints over 1,588 cM, while our AI-RIL captured 1,494 breakpoints. Four explanations might account for this difference. First, our cross design did not achieve the maximum potential of an AI-RIL design because exchange of worms between the pools of intercrossing worms was not performed as in [15]. Second, we genotyped fewer lines (167 compared to 236). Third, pervasive selection against AF16 alleles that occurs over much of the genome in the AI-RIL might have caused rapid reduction of heterozygosity during line construction prior to inbreeding, resulting in fewer observable recombination breakpoints. Finally, any contribution of self-progeny to the mating pools during the sib-mating phase of line construction, for example matings of male cross-progeny with hermaphrodite self-progeny, would reduce the map length. Although several lines contained one or more chromosomes with no apparent recombination breakpoints, none lack AF16 alleles completely. We can thus be certain that no lines were inadvertently established wholly from self-fertilization. Despite these potential issues, our AI-RIL cross scheme was successful at improving the resolution of the *C. briggsae* genetic map length compared to the previous F2 RIL-based version, capturing approximately twice the number of recombination breakpoints (Table 2).

Because the X chromosome is hemizygous in males during outcrossing (Figure 1), its map length in our design is expected to be 2/3 the length of the autosomal maps. Indeed, the expanded AI-RIL X map length, 100.0 cM, is similar to the expected value of 110.5. Unexpectedly, however, significantly fewer than expected SNPs were genotyped on the X. Although we cannot rule out the possibility that the *C. briggsae* X chromosome has reduced SNP density compared to autosomes, the method by which SNPs were chosen for genotyping is the most likely cause (Materials and Methods). Because only two markers are required both to order and to orient each supercontig within a chromosome assembly, chromosomes with larger supercontigs would have had fewer total SNPs genotyped per unit of length. Indeed, supercontigs assigned to ChrX are significantly larger on average than autosomal ones (t test, *P* = 0.02448; Figure S1), a possibility that had been noted earlier [38].

### An Improved *C. briggsae* Genome Assembly

The increased marker coverage of our genetic map allowed the incorporation of previously-unassembled genomic sequence supercontigs into the chromosomal assemblies and facilitated the genetic orientation of many supercontigs that were previously not oriented. Additionally, inconsistencies between the cb3 assembly [38] and the cb25 physical map [37], as well as three previously reported issues with cb3, have been resolved (Text S1).

The *C. briggsae* genome assembly is more complete than some recently-sequenced insect genomes, such as for *Nasonia vitripennis* [66], whose genome assembly comprises 63.6% of 312 Mbp of sequence based on a genetic map with more markers (1,255) but greater average inter-marker physical distance (249 kbp) [65]. The cb4 assembly now surpasses the *Drosophila melanogaster* genome assembly in completeness as well (version R5.33, flybase release FB2011_01 [67]). While 13.4% of the *D. melanogaster* genome sequence is unordered (half comprising unordered sequence from heterochromatic regions), the unordered content of *C. briggsae* has decreased from 15.9% (cb3) to 3% (cb4). However, compared to *C. elegans*, whose genome assembly is truly complete (*i.e.* containing no unordered sequence contigs, no gaps, and no uncalled bases), much work remains to complete the assembly of *C. briggsae*. The absence of heterochromatic centromeres and heteromorphic sex chromosome likely accounts for the relatively high quality of the Caenorhabditis assemblies.

### Interspecies Evolution of Recombination Rate in Caenorhabditis

Inter-species variation in recombination rate has been described in other taxa. In Helianthus, most intervals tested exhibited rate variation between species 0.75–1 million years (MY) diverged [68]. Variation among some Drosophila species also exists [69], but fine-scale recombination rates do not differ between others, suggesting lineage-specific and/or scale-dependent recombination rate variation [70]. Comparison of the *C. elegans* [15] and *C. briggsae* AI-RIL genetic maps reveals both conservation and variation in physical and genetic lengths of some recombination domains (Table 3). In both species, chromosome arms are clearly distinct domains that experience the vast majority of recombination events, and the distributions of arm recombination rates overlap, ranging from ∼2.5–8 cM/Mbp for autosomes. *C. elegans* arms tend to have slightly higher rates than *C. briggsae*, but *C. elegans* chromosomes also tend to be slightly smaller, so the elevated recombination rates likely reflect the necessity of fitting obligate recombination events into a shorter physical space.

Poor local synteny in the arm domains (Figure 3) prevented their direct comparison between species. We therefore compared the ratios of attributes for the two arms of a given homologous chromosome, assuming that aspects of the domains might be conserved despite mixing of the sequence content. For the AI-RIL-based genetic maps of both species, the ratios of arm physical or genetic lengths only exceeded two in one case, for the arm genetic lengths of *C. elegans* ChrI. The ratio of recombination rates of arms also occupied the same range, only once exceeding two (*C. elegans* ChrIV). However, this similarity should be interpreted carefully given the extent of intraspecies variation discussed below.

An additional *caveat* to the interpretation of the genetic parameters (map length and recombination rate) of the domains is that the values reported (Table 3) do not reflect recombination alone. Homozygosity resulting from selection acting on an allele during RIL construction would prevent the detection of future recombination events occurring in the domain and cause a deviation in the fixation of parental alleles in regions under selection. Evidence of such selection exists for chromosomes in *C. elegans* [15], [29]. In our *C. briggsae* AI-RIL, MTRD on ChrIII, ChrIV and ChrV also likely signifies the action of selection (discussed below). The regions experiencing MTRD are broad (Figure 2), but the arm whose allele fraction comes closest to the neutral expected value (IIIA, IVB, VB) is always genetically longer than the opposite arm. This matches the prediction that MTRD, possibly due to selection, results in a decrease in apparent recombination breakpoints and thus a reduction in genetic map length over part of a chromosome. In sum, each autosome exhibits a signature of selection, MTRD, in one of the two species. For this reason, the genetic values reported in Table 3 (both genetic length and recombination rate) might not represent the neutral recombination rate, especially for *C. elegans* ChrI and ChrII and for *C. briggsae* ChrIII, ChrIV and ChrV.

In contrast to map lengths, comparisons of physical attributes do not suffer from the influence of selection. The low recombination center domains, which have maintained greater synteny (Figure 3) over the roughly 18 MY since the common ancestor of *C. elegans* and *C. briggsae* [71], also revealed some size variation. Our findings concur with those from *C. elegans*, that the center domains are not precisely centered physically on the chromosome [15]. We find that, of the domain features tested, the proportion of total chromosome physical length occupied by the center domain is the most correlated between the species, suggesting that some aspect of relative physical position on the chromosome influences the positions of the center/arm domain boundaries.

### Intraspecies Evolution of Recombination Rate in *C. briggsae*

Work in a number of taxa has shown that recombination rates can vary within a species. A recent study of the evolution of recombination rates within mice found evidence for widespread rate differences among members of the species complex across 19% of the genome [72]. A remarkable seven-fold difference in recombination fraction within a Drosophila species has been revealed [69], and a detailed study of maps from intraspecific crosses in Nasonia revealed a slight (1.8%) but statistically significant increase in recombination frequency compared with interspecific crosses on a genome-wide scale [73]. Our findings from *C. briggsae* fall in the middle of this range, with the apparent recombination rates in homologous arm domains varying up to 2.9-fold between the crosses.

Our AI-RIL and F2 RIL paired parental strains between and within, respectively, *C. briggsae* clades that are estimated to have diverged about 90,000 years ago [26]. Examination of Figure 4 reveals that, for some chromosomes (ChrII and ChrX), the genetic lengths of both center and arm domains are constant. In addition, for each chromosome, the arm with the larger AF16/HK104 genetic map length is always the genetically larger arm in the VT847×AF16 map. However, substantial divergence in the genetic lengths of both the center domains (ChrIII and ChrIV) and arm domains (ChrI, ChrIV and ChrV) exists. The most striking feature of the genetic map comparison is the divergence in arm length ratio for multiple autosomes in the VT847×AF16 F2 RIL (Figure 4). Taken at face value, these results suggest that recombination itself is unusually biased to one arm in this cross, but alternative explanations should be considered. For example, we did not quantitatively compare our VT847×AF16 F2 RIL Marey maps to those previously reported for AF16×HK104 F2 RIL [38] because of the many differences in genome assemblies and markers scored in the two studies. Instead, we used our AF16/HK104 AI-RIL maps for the inter-strain comparison. However, both AF16/HK104 maps exhibit symmetrical arm usage, and generally resemble each other (except for total genetic length) more than either resembles the VT847×AF16 F2 RIL map. This suggests that intra-species differences are not caused by an artifact related to comparison of different cross designs.

Strong selection against individual loci or recombinant haplotypes could also account for asymmetrical apparent recombination rates in the two arms. However, evidence for both of these is lacking for the chromosomes that have arm genetic length ratios >2 (ChrI, ChrIV, and ChrV; Figure 4). First, the strong effect of genetic drift in the F2 RIL implies that any hypothetical deleterious recombinant genotypes would have to be severely debilitating to strongly bias breakpoint capture to one arm, yet no class of morbid progeny was observed during line construction. Also, no strong MTRD is evident in the F2 RIL (Figure S2), suggesting an absence of selection on individual loci. We therefore conclude that real differences in recombination are the most likely explanation for the asymmetric arm breakpoint capture in the VT847×AF16 F2 RIL. This suggests that recombination rate can vary over short periods of time but does not necessarily correlate with genomic divergence.

Greater variation in broad-scale recombination rate within rather than between species has also been observed in Nasonia [73]. The diversity in rate among populations of *C. briggsae* was unexpected, particularly given the similarities in the above interspecies comparisons and previous assertions that the overall similarity of recombination pattern among species likely reflects conservation [25]. Our results suggest that although the physical sizes of high and low recombination domains are stable within *C. briggsae*, variation in the degree of bias in usage of one arm over another exists. Comparisons with more genetic maps from other *C. briggsae* and *C. elegans* strains will likely reveal more diversity and patterns relevant to the understanding of the forces shaping the evolution of recombination rate.

The comparison of *C. briggsae* genetic maps also revealed three blocks of markers with inverted genetic order relative to flanking markers in one cross (Table S2). Because the AI-RIL and F2 RIL genetic maps share one parental strain, a physical difference in marker order in one of the strains, for example by physical inversion, would not be expected to produce this genetic effect. Possible explanations for this discrepancy include multiple recombination events that accumulated in a small physical interval and resulted in inaccurate estimations of genetic positions, or unappreciated copy number variation that created genotyping artifacts. However, a similar local reversal of marker order was observed in a study describing the behavior of genetic markers associated with polymorphic inversions in *Anopheles gambiae* [49].

### Factors Influencing Crossover Distribution

The stereotyped recombination domains for each linkage group have stimulated investigations into factors that might dictate their boundaries. Repeat density correlates with the domain structure [38] and is also associated with recombination rate differences in other species [72]. Likewise, inspection of Figure 3 suggests that many recombination domain boundaries are associated with loss of synteny. This finding suggests that local signals direct the locations of boundaries [15]. However, for both repeat content and synteny, it remains unclear whether these are causes or consequences of domain differences.

The molecular basis of the distribution of meiotic crossovers is only beginning to be understood. In *C. elegans*, DPY-28 acts in a classical condensin I complex to regulate the number and distribution of crossover events [74], [75]. In addition, loss of the chromatin protein XND-1 inverts the typical crossover distribution so that recombination occurs more frequently in the centers of chromosomes than in the arms [76]. Histone modifications on the arm and center domains are also distinct [77], suggesting an interplay between nucleosomes, condensins, and recombination in Caenorhabditis.

*C. elegans* chromosomes contain pairing centers: regions that promote homolog pairing and synapsis [78]. It has been suggested that these features might themselves have a *cis* effect on the distribution of recombination events, although their genetic locations in *C. elegans* do not perfectly correlate with recombination domain features [15]. Pairing centers might promote recombination in their vicinity, but this hypothesis cannot yet be tested in *C. briggsae* because no pairing centers have been characterized. Site-specific, perhaps *cis*-acting, segregating recombination rate modifiers, as are thought to exist in *C. elegans* [15] and mice [72], might also be responsible for observed variation. This might explain why variation in the extent of arm recombination asymmetry in the F2 RIL is restricted to a subset of chromosomes (Figure 4).

### Evolution of Genome Structure in Caenorhabditis

An earlier comparison of the *C. elegans* genome with *C. briggsae* assembly cb3, based on the positions of orthologous genes, revealed that the vast majority of rearrangements during divergence of these species were intrachromosomal and that syntenic blocks are larger on the X than on autosomes and also larger in center domains than on arms [38]. Our comparison using the cb4 assembly (Figure 3) qualitatively agrees with these previous findings. Specifically, syntenic blocks are longer in the low-recombining chromosome centers and are reduced or absent on the arms; the X chromosome exhibits the most structural similarity between the species. The relatively few off-diagonal sequence alignments (Figure 3) confirm the rarity of interchromosomal gene movement. We find no evidence of large interchromosomal translocations, although sequence divergence between *C. elegans* and *C. briggsae* might have obscured some that did occur.

Although the ortholog content of chromosomes is generally conserved (Figure 3, [38]), inter-arm movement has greatly eroded arm synteny between *C. elegans* and *C. briggsae*. Even the better-conserved center domains of chromosomes lack strict co-linearity. As a result, the relative orientation of the genetic and sequence maps of *C. elegans* and *C. briggsae* is basically arbitrary (Figure 3), especially for ChrII and ChrIII. The similarity of the recombination profiles of the chromosomes is therefore quite striking, reinforcing the impression that something other than gene content dictates the positions of recombination domain boundaries.

The comparison of two distinct *C. briggsae* genetic maps allowed us to ask whether the genetic signature of inversions exists. The strongest candidate region, within the B arm of ChrIV, provides the first genetic evidence of inversions distinguishing strains of *C. briggsae*. In this case, we conclude that an inversion of at most 666 kbp in HK104 relative to AF16 and VT847 likely exists. Given the hundreds of presumed translocations and/or inversions evident from the *C. elegans* and *C. briggsae* comparison (Figure 3) and the approximately 18 MY of divergence between the species [71], it is reasonable that a rearrangement distinguishing strains occurred during the divergence between the temperate and tropical clades of *C. briggsae*. The spacing of markers common to both the AI-RIL and F2 RIL genetic maps suggests that inversions up to 1 Mbp in size would often be undetectable in our analysis (particularly on the X chromosome). As in mice [72], it is possible that inversions unique to one strain or species are responsible for some of the recombination rate variation evident within and between species.

### Possible Causes of Marker Transmission Ratio Distortion

Large regions on ChrIII, ChrIV and ChrV in the AI-RIL preferentially fixed HK104 alleles to a degree not explained by sampling error alone (Figure 2), and nearly two-thirds of all AI-RIL marker genotypes are homozygous for the HK104 allele. Unintentional selection operating on hybrid genotypes during the intercross phase of RIL production is the most likely explanation for this widespread bias. In principle, selection could begin to cause MTRD as early as the F1 generation if a heterozygote-by-cross direction effect exists, but is not a factor here because there was no competition between cross directions during line production. More relevant here, selection on hybrid genotypes starting in the F2 generation would bias the transmission of parental alleles. We provide corroborating evidence for such F2 selection against AF16 alleles on ChrIII.

A modest bias of ChrIII toward HK104 was also evident in AF16×HK104 F2 RIL (Figure 5G) [38], presumably due to the acute developmental delay described here, but no MTRD was observed on ChrIV or ChrV. Our study should be more sensitive to incompatibilities because recombinant genotypes had substantial opportunity to compete against each other, whereas for the F2 RIL individual F2 were isolated immediately. This would be expected to allow genetic drift to dominate over all but the most severe fitness effects, such as that on ChrIII. Additionally, the AI-RIL cross scheme produced smaller haplotype blocks, perhaps separating co-adapted complexes of linked genes and creating more maladapted combinations of alleles than in F2 RIL. The difference in cross schemes might also explain the higher extinction rate of AI-RIL lines compared to the F2 RIL (59 of 240 vs. 1 of 112 lines).

Selection against a subset of hybrid genotypes is commonly ascribed to the presence of Dobzhansky-Muller incompatibilities (DMI) that arise when loci diverge in two strains experiencing reduced gene flow between them [79]–[81]. MTRD in hybrid Caenorhabditis genomes might also occur based on physical attributes of chromosomes regardless of the genes residing in the biased regions. In *C. elegans* males, homologous chromosomes differing by as little as 1 kb in length can segregate with biased frequencies, with the larger homolog included preferentially into the nullo-X gamete [82]. Homolog sizes could diverge between *C. briggsae* strains by expansion or contraction of repetitive sequences, which comprise over 22% of the genome [37]. Additionally, *C. elegans* isolates exhibit extensive copy number variation [83], suggesting that *C. briggsae* strains might as well. Meiotic drive can also produce MTRD [84]–[86]. However, selection against delayed development is sufficient to explain the ChrIII bias (see below), and neither size-based assortment bias nor meiotic drive would explain the cross-specific MTRD observed on ChrIV and ChrV. Thus, while these phenomena might occur to some extent, we conclude that they are not a major factor in determining AI-RIL genotypes compared to selection.

### An Inter-Strain Genomic Incompatibility Involving ChrIII

The F2 developmental delay phenotype associated with ChrIII (Figure 5) indicates that AF16 alleles at one or more loci in the central domain are dysfunctional when homozygous in a hybrid background. Delayed animals were unlikely to have been chosen for the next generation of the AI-RIL cross scheme, and this might entirely explain the MTRD seen on ChrIII (Figure 2). The lack of extensive LD between this distorted domain and other autosomal regions (Figure 6) suggests it interacts with HK104 alleles at multiple loci. Neither the delay phenotype nor MTRD on ChrIII (Figure 5H) were apparent during production of the VT847×AF16 F2 RIL, suggesting that the incompatibility does not exist in this cross. The phylogenetic and geographic relationships of AF16, HK104 and VT847 match the expectation that incompatibilities are more likely to arise between more divergent strains [28], [87], [88].

The smaller genetic map length of ChrIII relative to other autosomes in the AI-RIL (148.6 cM *vs.* 164.5–173.2 cM) might be another consequence of strong selection on ChrIII, as rapid loss of AF16 haplotypes reduces the opportunity for additional recombination events to produce detectable breakpoints. The ChrIII locus (or loci) responsible for the developmental delay phenotype is unlikely to be the same region of ChrIII involved in interchromosomal LD. The maximum MTRD for ChrIII occurs at roughly 5 Mbp, while the region of maximal D′ is limited to a small portion at 12 Mbp that also contains an unusual divergence of parental allele fixation between the two cross directions (Figure 2).

### Cross Direction–Specific Epistatic Interactions

Although all autosomal loci in the F1 founders of the AI-RIL are heterozygous AF16/HK104, cross direction alters the source of maternal cytoplasm and ChrX allele frequencies (Figure 1). These genetic distinctions between cross directions raise the possibility that an epistatic interaction between autosomal and either X chromosome or mitochondrial genome (mtDNA) alleles in a hybrid might cause MTRD on that autosome in only one cross direction, as seen on ChrIV and ChrV (Figure 2). If the mitochondrial and nuclear genomes have co-evolved through compensatory changes [89], DMIs might be revealed when two strains or species hybridize [90]. In hybrid AI-RIL, cytonuclear epistasis might cause preferential transmission of the autosome involved that originated from the parental hermaphrodite. Negative cytonuclear epistatic interactions might eventually produce reproductive isolation [91], although it has been argued that incompatibilities will rarely lead to the formation of independent species [92].

Such a model of cytonuclear coadaptation fits the pattern of MTRD on ChrIV in AF16×HK104 AI-RIL. These lines contain HK104 mtDNA and are overrepresented for ChrIV HK104 alleles (Figure 2). ChrX could also drive this bias, but the lack of LD between ChrX and ChrIV rules out this possibility (Figure 6). A coadaptation model cannot explain the biased fixation of HK104 alleles on ChrV in the HK104×AF16 AI-RIL (Figure 2), which bear AF16 mtDNA. A plausible alternative model here is cytonuclear transgressive segregation, in which a synergistic interaction between the mtDNA of one strain and a nuclear allele of the other produces fitness greater than either parental strain [93]. Consistent with this, we again see no evidence of LD between ChrV and ChrX (Figure 6). We therefore favor cytonuclear epistatic interactions (either coadaptive or transgressive) as the most likely explanations for the cross direction-specific MTRD on ChrIV and ChrV.

Other studies have reported similar patterns of MTRD in hybrid crosses. In Mimulus, an interpopulation cross exhibits MTRD involving multiple linkage groups [94], and in an interspecies cross, bias against the maternal genotype is seen [95], much like the pattern of bias on *C. briggsae* ChrV (Figure 2) that we tentatively attribute to transgressive segregation of mitochondrial and nuclear loci. Such patterns of MTRD are often attributed to cytonuclear incompatibility (*e.g.* in Nasonia wasps [96] and a moss [97]). Further, regions exhibiting MTRD might be expected to overlap the positions of hybrid incompatibility loci, as found in a cross between Solanum species [98]. However, it is unclear at what point (i.e., at what allele fraction threshold) an interchromosomal epistatic interaction might be classified as an incompatibility. Only when two incompatible loci are tightly linked, such as in the case of the *zeel/peel* lethal system on *C. elegans* ChrI, would allele fraction values be expected to approach unity. Even in that case, the allele fraction of linked markers in *C. elegans* AI-RIL do not reach unity [29]. Given the limited evidence for the presence of an extreme (*i.e.* lethal) incompatibility between AF16 and HK104, at this point we conclude only that cytonuclear epistatic interactions are responsible for the MTRD on ChrIV and ChrV. This is further supported by the significant difference between allele fraction values for the two cross directions in a block of markers on ChrV (Figure 2, asterisk).

### Plausible Mechanisms for Cytonuclear Epistatic Interactions

The nuclear genome encodes mitochondrial proteins, some of which interact with mitochondrion-encoded proteins involved in oxidative phosphorylation [90], [99]. The mitochondrial genome can co-adapt both with the nuclear genome [99] and with temperature [90], [100], and some hybrids in other taxa suffer from decreased oxidative phosphorylation efficiency [99], [101]. The mitochondrial genome of *C. briggsae* evolves rapidly [27] and is polymorphic for large deletions [102]. As this degree of mtDNA variation can impact fitness [27], [103], we propose that cytonuclear epistasis between AF16 and HK104 becomes evident when the mitochondrial genome is separated from co-adapted nuclear genes and/or provided nuclear alleles from a different strain. Similar incompatibilities have been discovered between many species (*e.g.* [104]–[106]) and can have complex genetic architecture [107]. Incompatibilities, cytonuclear or not, can contribute to speciation when hybrid fitness is sufficiently reduced [91], [108], [109].

### Potential Role of Local Adaptation in Marker Transmission Ratio Distortion

Fecundity in Caenorhabditis can be affected by temperature [110], and the strains employed in this study experienced substantially different temperatures in nature. Strain AF16 was isolated in Ahmadabad, India, a lowland tropical city (23°N latitude) where the average annual temperature is over 30°C (http://www.fao.org/countryprofiles/Maps/IND/07/tp/index.html). In contrast, HK104 was isolated in Okayama, Japan, a more temperate locale (34°N latitude) with an annual mean temperature of only 14°C (http://www.data.jma.go.jp/obd/stats/data/en/smp/index.html). Our AI-RIL were raised at 20°C, a temperature possibly more optimal for temperate strains [111]. Thus, the bias for HK104 alleles (61% of genotypes) in the AI-RIL might reflect selection for temperature-adapted genes. Furthermore, although 120 lines in each cross direction were initiated, only 95 AF16×HK104 and 86 HK104×AF16 lines survived. Line extinction might reflect selection against hybrid genotypes specifically unsuited to 20°C. Repetition of the hybrid crosses at higher temperatures might yield different results, yet at 20°C under lab conditions, HK104 individuals produce fewer offspring over their lifetime than AF16 [110], [112]. This suggests that a temperature-dependent effect separate from total fecundity might explain the bias of HK104 alleles in the AI-RIL. Alternatively, line extinction might be due to generalized outbreeding depression between the strains [113]. The regions of significant MTRD coincide with the central recombination domains (Figure 2) and associated blocks of LD (Figure 6). Thus, selection on loci in the central domain, which will rarely be separated by recombination, can affect the population genetics of half of a chromosome [114]. While the recombination profile of Caenorhabditis chromosomes amplifies the population genetic signals of selection, the near-absence of recombination in the central domain is an obstacle to fine-scale mapping of loci under selection.

### Future Prospects

The genotyped AI-RIL described here serve as a powerful new resource for the mapping of divergent phenotypes, as has been accomplished using *C. elegans* RIL [35]. For example, they are being used to explore the genetic architecture of temperature tolerance of AF16 and HK104 (A. Cutter, pers. comm.) To continue improving resources for the study of *C. briggsae*, future efforts should identify genetic markers on remaining unassembled sequence supercontigs in order to incorporate them into the genome assembly. Further increasing the marker density might also identify yet more misassemblies that exaggerate the apparent genomic divergence between *C. briggsae* and related species.

More biologically, we note that the genetic structuring of *C. briggsae* strains by latitudinal zone [17], [25]–[28] is not seen in *C. elegans*. Whether the epistatic effects described here represent maladaptive loss of local adaptations in hybrids or more generalized incompatibilities, only a few intra-species hybrid incompatibility loci have been described at the molecular level in animals (reviewed in [108], [109]). Future efforts will focus on mapping the hybrid developmental delay locus on ChrIII and testing the hypothesis that cytonuclear epistasis exists among *C. briggsae* strains diverged roughly 100,000 years [26]. It has been known for some time that some species of Caenorhabditis are cross-fertile but post-zygotically reproductively isolated [115]–[118]. The recent identification of fertile interspecies hybrids between *C. briggsae* and *C.* species 9, which shared a common ancestor as recently as one million years ago [26], has facilitated the study of post-zygotic reproductive isolation [119]. Thus, *C. briggsae* provides unique opportunities to explore different stages of reproductive isolation in the nematode phylum.

## Materials and Methods

### Strains and Lines

Advanced-intercross recombinant inbred lines (AI-RIL) were produced from the *C. briggsae* strains AF16 from Ahmadabad, India [33] and HK104 from Okayama, Japan (H. Kagawa). Crosses between males and sperm-depleted hermaphrodites were established in both directions, and several mated (as determined by presence of a copulatory plug) hermaphrodite F1 produced a large F2 population. Three plugged F2 hermaphrodites (each having mated with one or more males) were chosen to found 120 lines from each cross direction. Generations F3–F7 were similarly founded by a population of three plugged hermaphrodites. The exact relatedness between mates thus varied, but should have been no closer than biparental full-sibs. During the F3–F7 generations, matings would have occurred between progressively more restricted genotypes, such that by F8 substantial homozygosity might have already existed. From F8–F17, the lines were intentionally inbred by complete selfing using a single virgin (L4 stage) founder hermaphrodite per generation. 95 lines were produced for the AF16×HK104 cross (male×hermaphrodite), and 86 for the HK104×AF16 cross. The disparity between the number of lines initiated and that produced was due to the extinction of lines. Additionally, one AF16×HK104 line was not genotyped.

F2 RIL were produced from AF16 and the *C. briggsae* strain VT847 from Hawaii [30]. Crosses between VT847 males and sperm-depleted AF16 hermaphrodites were performed as described [38]. Eighty-nine RIL were initiated from individual F2 hermaphrodites produced by sib-mated F1 individuals, then inbred by one L4 hermaphrodite per generation through F11.

DNA was extracted from AI-RILs with a QuickGene-Mini80 using the DNA tissue kit S (Fujifilm Corp., Tokyo, Japan).

### Genotyping and SNP Distribution

The genotypes of 180 AI-RIL, 93 F2 RIL, and parental strains were obtained using the GoldenGate genotyping assay (Illumina, [120]). The DNA samples were genotyped with 1,536 single nucleotide polymorphism (SNP) marker assays distinguishing AF16 from HK104 and/or VT847 [39]. These SNP markers were chosen 1) on the basis of their distribution on sequence supercontigs in order to genotype at least one marker on as many of the largest supercontigs as possible, and also 2) to maximize the number of large supercontigs containing at least two markers, so that the supercontigs could be oriented. Because the chromosomal assignment of supercontigs containing the markers was not considered during marker selection, the genome-wide distribution of genotyped SNPs was expected to reflect the true distribution of SNPs. Autosomal and X chromosome supercontig lengths were analyzed via var.test and an unpaired two-sample t test in R.

Genotypes of pools of delayed F2 hybrids were determined through sequence analyses of PCR amplification products derived from *Cbr-egl-5* and *Cbr-mab-20*. Forward and reverse primers for *Cbr-egl-5* were (5′ to 3′) CCGAGATTCAGAAAACCCGAAG and CACTACAGTAAACCCCCTCAAGACC, respectively. Forward and reverse primers for *Cbr-mab-20* were TGCTCTTCGGTTGGAATGCGAC and CGGTTTTTTGGTTTGATGGTGGG, respectively. Sequencing reactions for both genes were primed with the forward primers.

### Analysis of Genotype Data

Raw GoldenGate assay data were analyzed with GenomeStudio 2008 (v. 1.0.2.20706) using the genotyping module (v. 1.0.10, Illumina). The data were required to exceed the following quality control thresholds in order to be analyzed. Numbers in parentheses represent the number of samples or assays not exceeding each threshold in the AI-RIL.

#### DNA samples: mean R-value>0.5 (0)

Thirteen of the 180 genotyped AI-RIL, which had >5% heterozygosity, were manually removed because it was empirically determined that their presence in the dataset confounded robust estimation of genetic maps. Data from the remaining 81 AF16×HK104 and 86 HK104×AF16 AI-RIL were used in our analyses. 89 of the VT847×AF16 RIL passed these quality control thresholds and were used in our analyses.

#### SNP assays: GenTrain score >0.4, call frequency >0.95, and mean R-value>0.2 (328)

The boundaries of genotype clusters were then hand-edited because GenomeStudio expects Hardy-Weinberg equilibrium, which is a condition violated by selfing organisms. As a result, more genotype calls than expected for our cross design were initially assigned as heterozygote by GenomeStudio.

For the AI-RIL data, monomorphic markers (115) were excluded, as were markers for which both parental strains were assigned the same genotype (2). 59 more assays were removed due to weak clustering of genotype calls, low R values only in one genotype cluster, or presence of >5% heterozygous calls, a condition in which it was often impossible to distinguish whether the assay failed or whether these were valid data. Data from the remaining 1,032 SNP markers were used in our analysis.

For the VT847×AF16 F2 RIL, 209 assays passed the quality control thresholds, the large proportion of excluded assays largely due to monomorphism in these two strains (data not shown).

### Genetic Map Production

The 172,344 AI-RIL genotype calls (Table S1) were imported into Map Manager QTXb20 (v. 0.30) [121]. A genetic map for each of the six linkage groups (five autosomes and the X chromosome) was estimated using the following parameters: probability of incorporation into a linkage group 1×10^−6^, Haldane map function, and intercross linkage evaluation. The cb3 map, produced from F2 RIL, was estimated using self-RI linkage evaluation [38]. However, this approach infers per-meiosis recombination rates from breakpoints accumulated over multiple generations, and thus reports compressed map lengths inconsistent with the number of observed recombination breakpoints in the AI- RIL. Selecting intercross evaluation, similar to the approach of selecting backcross evaluation to estimate AI-RIL maps in [15], forces Map Manager QTXb20 to regard all breakpoints as occurring in a single meiosis. The resulting longer map lengths reflect the numbers of recombination breakpoints observed (Table 2) and are thus more directly comparable to other AI-RIL maps.

Map Manager QTXb20 was also used to estimate genetic maps using the 18,601 VT847×AF16 F2 RIL genotype calls (Table S1) with the same parameters as previously used for *C. briggsae* F2 RIL [38]. A strategy of relaxation of the probability of incorporation was employed to incorporate five markers into the six major linkage groups, as in [38]. As was the case for the AI-RIL, it was empirically determined that the presence of 50 heterozygote genotype calls prevented robust map estimation. Therefore, these calls were considered as missing data in Map Manager QTXb20 and are reported as such (“?”) in Table S1.

Map Manager QTXb20 reported the numbers of recombination breakpoints per linkage group used to calculate average breakpoint capture (Table 2). However, because it does not count breakpoints associated with heterozygote calls under self-RI linkage analysis, the counts were manually increased to account for breakpoints necessary to produce heterozygote genotypes.

We noticed an artifact introduced when map positions were calculated using Map Manager QTXb20: map positions were offset by one marker. Exports of some linkage maps gave the genetic position of the first marker in the map as non-zero; the position of the last marker in each map was never reported, and the last marker in any block of non-recombinant markers was always reported to have a map position different from the others in that block. Defining the position of the first marker in each linkage group as 0 centimorgans (cM) and then shifting each subsequent map position by one marker resolved these discrepancies. This artifact might explain why some markers in the cb3 linkage maps are nonrecombinant yet flank haplotype breakpoints and differ in allele fraction: the reported genetic positions of the markers might differ slightly from their true values. The orientations of linkage maps produced in this study were compared with the cb3 maps [38] and inverted when necessary to maintain the same relative map positions of markers.

### Genome Reassembly

Based on our new genetic maps and the locations of the SNP markers on sequence supercontigs, we first reassembled the genome from the cb25 supercontigs [37] and then compared this assembly with cb3 [38]. For a few supercontigs (see Text S1), the cb3 genetic maps contained more information than the cb4 maps. In these cases, we supplemented our data with data from cb3. Only where our data contradicted or improved upon the cb3 assembly did we make changes. Where necessary, cb25 supercontigs were split to resolve discrepancies between the genetic and physical order of markers (see Text S1). Figure S3 depicts the decision tree employed to resolve these discrepancies; the genetic and physical map data used to select locations at which to split supercontigs to resolve certain discrepancies are provided in Table S3. Genome assembly version cb4 is available at http://www.wormbase.org.

### Definition and Normalization of Recombination Domains

Each tip domain (two per chromosome) comprises the sequence between a chromosomal assembly terminus and the most internal genetic marker in the terminal block of non-recombinant markers. By definition, these domains have a recombination rate of zero. For the AI-RIL, the boundaries of the arm-center recombination domains were identified by segmented linear regression for each linkage group as in [15] using the “segmented” package implemented in R [122].

The genetic map positions of recombination domain boundaries were estimated for the AI-RIL by linear interpolation from the two markers flanking each boundary. The lower marker density in the F2 RIL genotype data set reduced confidence in the accuracy of boundaries estimated by segmented linear regression, so we imposed the physical positions of domain boundary estimates from the AI-RIL onto the F2 RIL genetic maps and estimated the genetic length of each domain as above. The recombination rates for *C. elegans* domains reported in Table 3 differ from those previously reported [15], which were rate estimates based on the slopes of segmented linear regression. Here, we calculated *C. elegans* domain genetic lengths, as above, from the interpolated genetic positions of domain boundaries (kindly provided by M. Rockman, unpublished; Table 3).

To facilitate comparison between maps, we used a unique correction factor for each linkage group to normalize the sum of estimated genetic lengths of the three domains to 50 cM, the expected per-meiosis length under selfing.

### Evaluation of Inter-Species Chromosomal Synteny

*C. elegans* (release ws185, the assembly version used to define recombination domain boundaries in [15]) and *C. briggsae* (cb4) genome sequences were first masked using RepeatMasker 3.2.9 with default parameters and the June 4, 2009 RepBase repeat libraries [123]. The masked sequences were then compared with MUMmer 3.22 [124] using nucmer to identify only maximal unique matches.

### Marker Transmission Ratio Distortion Analysis

We compared the observed number of AI-RIL fixed for the HK104 allele to the expectation of 50% with a Bonferroni-corrected chi-square test. Because linked markers are not truly independent tests, the effective number of independent tests was estimated as follows: The autocorrelation parameter at lag = 1 was estimated for the allele fraction data within each recombination domain for each cross direction using the acf() function in the base package of R. The value of the autocorrelation parameter was then used to estimate the effective number of tests [125]–[127]. The significance threshold p = 0.05 was then Bonferroni-corrected by the genome-wide sum of effective number of tests for each cross direction and used to calculate the allele fraction value, plotted in Figure 2, at which a marker would reach genome-wide significance for deviation from the expected value.

To test for epistasis between cross direction and the ChrIV or ChrV center domain markers, the allele fraction values for both cross directions were compared using Fisher's exact test in R. The significance threshold p = 0.05 was then Bonferroni-corrected by the sum of the largest effective number of tests estimated above for the two center domains for both cross directions.

### Linkage Disequilibrium

After identifying the relative genetic order of markers, the genotype data from each AI-RIL cross direction were imported separately into Haploview v. 4.2 [128]. With the Hardy-Weinberg p-value cutoff set at 0, intra- and inter-chromosomal linkage disequilibrium D′ values were plotted using the Standard color scheme (Figure 6). One pair of markers exhibiting D′> = 0.8 from each block of markers in interchromosomal LD was selected to test for significance using the chi-square test. Expected counts of AI-RIL fixed for the same parental allele at two loci were calculated according to the parental allele frequencies at each locus for each cross direction.

## Supporting Information

Figure S1 Shotgun assembly supercontig size distribution for autosomes versus X. Each panel is a histogram depicting the number of supercontigs (sctg) in 0.5 Mbp size bins for the indicated set of supercontigs. The greater mean of the X-linked distribution is significant (see Discussion).
(EPS)

Figure S2 Allele fraction plots for VT847×AF16 F2 RIL. For each chromosome, the fraction of lines fixed for the VT847 allele (black line) at each marker is given. Vertical lines indicate marker positions, which are plotted on the X-axis (chromosomal assembly position in Mbp). For each chromosome, the expected value of allele fraction is 0.5.
(EPS)

Figure S3 Decision tree used to identify unambiguous sites for supercontig splits. Input is shown in the rounded rectangle at top, with the total number of splits required to resolve all genetic-physical map discrepancies given in parentheses. Questions leading to dichotomous decisions are shown as diamonds. Circles contain decisions, where parentheses show the number of splits made. Numbers beneath the circles reference the cb4 splits created by each decision (Table S3). The shaded circle deals with cb3 supercontig splits removed in our assembly. Because these cases do not represent the creation of splits, they are not part of the input to the decision tree but are shown in order to enumerate all changes to supercontig splits made between cb3 and cb4 also shown in Table S3. Descriptions of decisions other than “use site”: Choose site by interpolation: where a split was necessary to allow insertion of another supercontig, if the genetic marker on that donor contig was recombinant with the markers flanking it on the recipient supercontig, the local recombination rate (cM/Mbp) between the flanking markers was calculated and the physical position of the marker on the donor contig was interpolated. The supercontig gap site closest to this position was chosen as the supercontig split site. Minimize physical distance: When a marker on a donor supercontig was nonrecombinant with one flanking marker on the recipient supercontig, making it impossible to interpolate the physical position of the donor marker, the supercontig gap closest to the nonrecombinant marker on the recipient contig was chosen as the split site. Make smallest sctg fragment: When a split was necessary to reorder or invert resulting supercontigs, if multiple supercontig gaps existed, the gap that would result in the production of the largest and smallest resulting supercontigs was chosen as the split site. We reasoned that this would be parsimonious with the process of FPC assembly: that removing the smallest amount of sequence from the FPC would be the least likely to alter the restriction fingerprint that was the basis for the cb25 physical map. Move discrepant ctg only: in the absence of any data that would suggest a split site, we moved only the sequence contig containing the offending marker by splitting on either side of the contig.
(EPS)

Table S1 Genome assembly and SNP genotypes. Columns in worksheet “AF16-HK104 AI-RIL” contain the names of the 1,032 SNPs used in the analysis, the name of the cb25 supercontig containing each SNP, the length of that supercontig, the chromosome assignment of the supercontig based on the genetic linkage map (Chromosome 6 is the X chromosome), the start and end coordinates of each supercontig within each chromosome assembly, the nucleotide position of the SNP within the supercontig and within the chromosome assembly, and the genetic map position (in Morgans) of the marker. These genetic positions have been corrected for a Map Manager QTXb20 (MM)-introduced artifact and oriented with respect to the map positions in the cb3 genetic map (Materials and Methods). The genotypes of the AF16 and HK104 parental strains and the AI-RIL are coded in ABH format: A = AF16/AF16, B = HK104/HK104, H = AF16/HK104. Columns in worksheet “VT847×AF16 F2 RIL” contain the names of the 209 SNPs successfully genotyped in the F2 RIL (bold are the 132 markers also typed in the AI-RIL), the genetic map positions (Map Manager QTXb20-corrected and oriented with respect to the cb3 genetic maps), their cb4 chromosome assignments and assembly positions. The genotypes of the RIL are coded in ABH format: A = AF16/AF16, B = VT847/VT847. The 50 genotype calls that were initially estimated to be heterozygote (AF16/VT847) are reported here as missing data (“?”) because of the likelihood that these are not accurate genotype calls (see Materials and Methods).
(XLSX)

Table S2 Analysis of possible inversions. Columns contain the SNP name, cb4 chromosome assignment, and cb4 chromosome assembly position of the 132 SNPs common to the AI-RIL and F2 RIL datasets. The genetic position (in centimorgans) of each marker in the AF16/HK104 and VT847×AF16 genetic maps, normalized to produce linkage groups of length 50 cM, is given. Blocks of markers that are nonrecombinant in one map but recombinant in the other, consistent with the expectation of an inversion, are boxed. Blocks of markers whose genetic order is inverted relative to the other map are shaded.
(XLSX)

Table S3 Analysis of genetic-physical map discrepancies. This table contains information about the *C. briggsae* genome physical map (cb25 assembly FPC and supercontig data [37], shades of gray), and the genetic and physical map positions of genetic markers genotyped to produce genetic map and genome assembly versions cb3 and cb4. All cb25 assembly data for only those supercontigs affected by cb3 or cb4 genetic-physical map discrepancies are shown in columns A–I; these data are necessary to identify the positions of unsupported supercontig gaps within FPCs at which FPCs can most reasonably be split (rows where Ctg Start="clone” and Ctg End="no”). The next six columns, containing cb4 data (shades of blue), provide the cb4 assembly coordinates of the supercontigs; the names, positions, linkage group (LG) assignments, and map positions of genetic markers typed in our study, a description of the action taken to resolve discrepancies between the physical order of markers (according to the cb25 sequence assembly) and their genetic order (based on the cb4 genetic map), and the single-letter suffix assigned to split supercontigs. The same information from cb3 [38] is provided (shades of orange) for comparison. For ease of referring to supercontig splits, we have numbered the cb3 splits (“cb3-1” to “cb3-30”) and the cb4 splits (“cb4-1” to “cb4-63”). In cases where a split was introduced within a supported sequence contig, the exact position of the split is given in the “Action” column. Row shading was employed to visually identify positions of supercontig splits. Aside from splitting, the other actions taken to resolve discrepancies were to move and invert supercontigs created by splitting relative to each other. Positions where cb3 splits were removed are also noted. Supercontig suffix rubric: when supercontig splits were made in cb3, sequential single-letter suffixes were added to the supercontig name (e.g. fpc0001a, fpc0001b) to name the supercontigs resulting from the split. We continue this practice here and have introduced a numerical annotation to allow cb3-split supercontigs that didn't change in cb4 to be easily distinguished from supercontigs that were amended in cb4. Where both cb3 and cb4 make entirely the same set of splits for a supercontig, the suffix remains the same in cb4 (e.g. for the supercontigs comprising fpc0010). If a cb3 split was removed in cb4, we no longer used the suffix corresponding to the second (“b”) supercontig. In cases where a supercontig containing a suffix in cb3 (e.g. fpc0071c) was further split in cb4, the largest cb4 supercontig resulting from new splits maintained the suffix of the cb3 supercontig but with a digit suffix (part of cb3 fpc0071c became cb4 fpc0071c2) and the remainder of the new supercontigs created were given new single-letter suffixes.
(XLSX)

Text S1 Details of the *C. briggsae* genome reassembly process.
(DOC)
