# Supplementary material for: Caenorhabditis briggsae Recombinant Inbred Line Genotypes Reveal Inter-Strain Incompatibility and the Evolution of Recombination
Source: PLoS Genet. 2011 Jul 14;7(7):e1002174. doi: 10.1371/journal.pgen.1002174 (PMC3136444; doi:10.1371/journal.pgen.1002174)
Supplement: Text S1 — Details of the C. briggsae genome reassembly process. (DOC) [file pgen.1002174.s007.doc]

**Text S1. Details of the *C. briggsae* genome reassembly process**

In the first genome assembly, version cb25, the whole-genome shotgun sequence was initially assembled into contigs on the basis of overlapping sequence reads. Contigs were then assembled into supercontigs on the basis of paired-end relationships. A physical genome map, based on restriction patterns of large-insert clones (a fingerprint contig or FPC map), was then used to assign sequence supercontigs, by means of *in silico* digests, to individual FPCs [1]. A physical map-based assembly is expected to contain errors because gaps between sequence supercontigs can obscure their order and orientation within an FPC.

In the assembly of version cb3, the cb25 supercontigs had been concatenated to create chromosome assemblies based on the relative positions of genetic markers within the sequence supercontigs and within the genetic map. Some discrepancies between the genetic order of SNP markers and their physical order in the cb25 FPC-based sequence assemblies had been noted [2]. Most genetic/physical map discrepancies in cb3 occurred between markers on different sequence supercontigs that had been assigned to the same FPC. In most of these cases, a discrepancy could be resolved by splitting the FPC at an unsupported gap between sequence supercontigs and changing the order or orientation of the resulting supercontigs. Twenty-seven such splits in unsupported gaps were reported in cb3 (though see below – the true number is twenty-eight for cb3); the remaining discrepancy required to be resolved by introducing a split within a sequence supercontig [2].

Our data failed to support one of the above splits made in cb3. For one on fpc2888, in which markers mapped to two linkage groups were contained on the same FPC, the discrepancy was resolved in cb3 by splitting the FPC in an unsupported gap. However, in this case there were multiple gaps between the markers assigned to different linkage groups, making the choice of split site ambiguous. In our data set, the higher density of markers eliminated all but one of these choices, a site different than that chosen in cb3. Importantly, for the within-supercontig split made in cb3 in fpc0065, our data do agree with the cb3 synteny-based analysis that suggested that two markers on the same supercontig belonged on two different linkage groups, confirming that errors in supercontig assembly, while rare, do exist in the cb25 assembly.

Altogether, 37 FPCs were split into 100 supercontigs to produce assembly cb4 (Table S3, Figure S3). During the process of SNP genotype quality control, we also noticed that the alleles of several markers contained on three supercontigs were completely concordant with the hermaphrodite parent’s genotype. This suggested that these supercontigs are from the mitochondrial genome, which was confirmed by BLAST [3]. We therefore removed these three supercontigs (NA_260, NA_264 and NA_292, totaling 20,708 bp) to produce the Cb4 nuclear genome assembly, which comprises 108,357,065 bp on 638 supercontigs (Table 1). 277 of these, comprising 105 Mbp, were also ordered along the six chromosome assemblies.

Eight supercontigs were mapped to a chromosome but unable to be unambiguously inserted into the chromosome assembly. One 29 kbp supercontig contains a marker mapped to a position on the X chromosome that implied the need to insert the supercontig within an existing FPC. However, no unsupported clone gaps exist in this target FPC, nor did a breakpoint in synteny suggest a specific insertion site. We therefore assigned the supercontig to ChrX, but not to a specific position (creating the “chromosome X random” assembly). In addition, six supercontigs contained single markers that were successfully genotyped in cb3 but not in cb4. For two of these (NA_025 and NA_031, totaling 59,421 bp), their locations relative to flanking supercontigs in cb3 remains unambiguous in the cb4 ChrV assembly and they were able to be included in chromosome assemblies. The other four supercontigs have ambiguous positions in the cb4 assembly and thus have been relegated to chromosome random assemblies. Three supercontigs were assigned to chromosome assemblies in cb3 yet lacked markers with reported genetic positions; these also lacked markers in cb4. These three supercontigs were also assigned to chromosome random assemblies in cb4.

353 small supercontigs, ranging in size from 1-85 kbp and comprising a total of 2.9 Mbp, were unassigned to chromosome assemblies for lack of genotyped markers on them and comprise the “unassembled” chromosome. Overall, 35 markers on 15 supercontigs now have different chromosome assignments than in cb3.

145 supercontigs (89,167,672 bp) are oriented in the cb4 assembly. 131 (85,124,118 bp) were oriented using the cb4 genetic maps, while orientation of five (1,599,986 bp) relied on data from the cb3 genetic map. For nine supercontigs (2,443,568 bp), neither the cb4 nor the cb3 maps were able to genetically orient the sequence, but we retained synteny-based orientations from cb3. Only in a single case (fpc4063) did the genetic orientation of a supercontig disagree between cb3 and cb4. We resolved this discrepancy in favor of cb4, which contained data from more markers on the supercontig. In three instances (fpc0024, fpc4140 and fpc4260a), synteny-based orientation of supercontigs in cb3 disagreed with genetic orientation in cb4. For these cases, the discrepancies were resolved in favor of the genetic orientations in cb4.

Genome assembly cb4 resolves several existing inconsistencies. One such discrepancy noted in [2], involving fpc3752, was resolved by moving the contig containing a single marker to the start of the supercontig. Similar misassemblies in cb25 first identified here resulted in 1.8 Mbp of sequence on 15 supercontigs changing chromosomal assignment from cb3 to cb4. In some cases, sequences from multiple chromosomes had been assembled into single FPCs in cb25; these were not detected in cb3 for lack of markers on each segment of sequence belonging to a different linkage group. Greater marker density in the cb4 map has also allowed genetic orientation of additional supercontigs within chromosomal assemblies. In several cases, the synteny-based orientations from cb3 did not agree with the genetic orientations from cb4, possibly because synteny in these regions does not actually exist. Another cb3 inconsistency was noted between the number of sctgs resulting from splits made (48) and the number of splits reported (28). To generate the 48 sctgs, 29 splits would be required. We noticed that fpc0071 had initially been split into four sctgs in cb3, but two of the resulting sctgs mapping adjacent to each other on the same chromosome had been rejoined without reporting one of the initial three splits. Thus, 29, not 28, splits to cb25 sctgs were made while producing cb3. The final inconsistency we noticed was that the sum of lengths of the two cb3 sctgs originating from fpc0065 (the lone case where a split within a sequence supercontig was made) did not match the original length of fpc0065. In this case, we discovered that a gap of 1000 N in cb25 had been changed to a gap of 100 N in cb3. We restored this gap to a length of 1000 in cb4. For this reason, cb3-fpc0065b is now cb4-fpc0065b2. Although the proximal breakpoint is the same in both assemblies, the addition of 900 N to cb3-fpc0065b means that the cb4 version technically differs (in length and sequence).

The new assembly also resolves three previously reported issues with cb3. First, in Figure 2 of Ref. [2], a break in synteny of orthologous ChrII genes in *C. elegans* and *C. briggsae* is evident. The genetic map produced here reveals that roughly 100 kb of sequence in fpc2454 should be inverted and moved to the start of the FPC, which restores ortholog colinearity. Given this and other breakpoints and rearrangements that the new genetic map suggests, previous estimates of *C. elegans* and *C. briggsae* divergence based on genome rearrangements might be less accurate than thought [4]. In addition, cb4 resolves two possible misassembly issues reported by Zhao *et al*. [5]. They noted that sequence from one end of ChrIII might belong at the opposite end of the chromosome; in our assembly, such a change in fpc4010 is made. Also, they suggested that a 125 kbp piece of ChrV should be moved to ChrIV. Indeed, our genetic evidence suggested that 124 kbp of fpc2220 on ChrV belongs on ChrIV.

**Literature Cited in Supplemental Text**

1. Stein LD, Bao Z, Blasiar D, Blumenthal T, Brent MR, et al. (2003) The genome sequence of *Caenorhabditis briggsae*: a platform for comparative genomics. PLoS Biology 1: E45.

2. Hillier LW, Miller RD, Baird SE, Chinwalla A, Fulton LA, et al. (2007) Comparison of *C. elegans* and *C. briggsae* Genome Sequences Reveals Extensive Conservation of Chromosome Organization and Synteny. PLoS Biology 5: e167.

3. Altschul SF, Gish W, Miller W, Myers EW, Lipman DJ (1990) Basic local alignment search tool. Journal of Molecular Biology 215: 403-410.

4. Coghlan A, Wolfe KH (2002) Fourfold faster rate of genome rearrangement in nematodes than in Drosophila. Genome Research 16: 857-867.

5. Zhao Z, Flibotte S, Murray JI, Blick D, Boyle TJ, et al. (2010) New tools for investigating the comparative biology of *Caenorhabditis briggsae* and *C. elegans*. Genetics 184: 853-863.
